# Low levels of fine particulate matter increase vascular damage and reduce pulmonary function in young healthy adults

**DOI:** 10.1186/s12989-020-00389-5

**Published:** 2020-11-16

**Authors:** Lauren H. Wyatt, Robert B. Devlin, Ana G. Rappold, Martin W. Case, David Diaz-Sanchez

**Affiliations:** Public Health and Integrated Toxicology Division, Human Studies Facility, United States Environmental Protection Agency (USEPA), Research Triangle Park, 104 Mason Farm Rd, Chapel Hill, NC 27514 USA

**Keywords:** Systemic inflammatory effect, Healthy human volunteers, Particulate matter air pollution, Controlled exposure

## Abstract

**Background:**

Fine particulate matter (PM_2.5_) related mild inflammation, altered autonomic control of cardiovascular function, and changes to cell function have been observed in controlled human exposure studies.

**Methods:**

To measure the systemic and cardiopulmonary impacts of low-level PM exposure, we exposed 20 healthy, young volunteers to PM_2.5_, in the form of concentrated ambient particles (mean: 37.8 μg/m^3^, SD 6.5), and filtered air (mean: 2.1 μg/m^3^, SD 2.6). In this double-blind, crossover study the exposure order was randomized. During the 4 h exposure, volunteers (7 females and 13 males) underwent light intensity exercise to regulate ventilation rate. We measured pulmonary, cardiac, and hematologic end points before exposure, 1 h after exposure, and again 20 h after exposure.

**Results:**

Low-level PM_2.5_ resulted in both pulmonary and extra-pulmonary changes characterized by alterations in systematic inflammation markers, cardiac repolarization, and decreased pulmonary function. A mean increase in PM_2.5_ concentration (37.8 μg/m^3^) significantly increased serum amyloid A (SAA), C-reactive protein (CRP), soluble intercellular adhesion molecule-1 (sICAM-1), and soluble vascular cell adhesion molecule-1 (sVCAM-1), 1 h after exposure by 8.7, 9.1, 10.7, and 6.6%, respectively, relative to the filtered air control. SAA remained significantly elevated (34.6%) 20 h after PM_2.5_ exposure which was accompanied by a 5.7% decrease in percent neutrophils. Decreased pulmonary function was observed 1 h after exposure through a 0.8 and 1.2% decrease in forced expiratory volume in 1 s (FEV_1_) and FEV_1_/ forced vital capacity (FEV_1_/FVC) respectively. Additionally, sex specific changes were observed in repolarization outcomes following PM_2.5_ exposure. In males, P-wave and QRS complex were increased by 15.4 and 5.4% 1 h after exposure.

**Conclusions:**

This study is the first controlled human exposure study to demonstrate biological effects in response to exposure to concentrated ambient air PM_2.5_ particles at levels near the PM_2.5_ US NAAQS standard.

**Clinical trial registration information:**

clinicaltrials.gov; Identifier: NCT03232086. The study was registered retrospectively on July 25, 2017, prior to final data collection on October 25, 2017 and data analysis.

**Supplementary Information:**

The online version contains supplementary material available at 10.1186/s12989-020-00389-5.

## Introduction

Exposure to particulate matter (PM) air pollution is associated with elevated rates of mortality and morbidity [1–4]. In 2015, it was estimated that over 4.2 million premature deaths and over 103 million years of healthy life lost were attributable to ambient particulate matter pollution, making it one of the top global health risk factors [5]. Similarly, the American Heart Association has concluded that “Exposure to PM <2.5 μm in diameter (PM_2.5_) over a few hours to weeks can trigger cardiovascular disease-related mortality and nonfatal events” [1]. Associations with short-term PM_2.5_ exposure have been demonstrated in more than 100 time-series and case-crossover analyses and include risk of a myocardial infarction (MI), aggravated asthma, changes in acute respiratory response, and increased risk for hospitalization and mortality [1, 6–12].

Findings from these epidemiological studies are strengthened by data from controlled human exposure studies where subjects are exposed on separate occasions to clean air or known PM_2.5_ concentrations and pollutant-induced changes in cardiopulmonary biomarkers are assessed post-exposure. Controlled human exposure studies have shown that healthy volunteers exposed to PM_2.5_ primarily exhibit mild pulmonary inflammation, altered autonomic control of heart function, altered endothelial cell function, vascular inflammation, and changes in blood factors associated with coagulation and fibrinolysis, when compared to clean air exposures [11]. Controlled exposure studies provide biological plausibility to epidemiological studies by delineating pathophysiological changes following PM_2.5_ exposure in relevant biological pathways that can explain the epidemiological findings. Unlike epidemiological studies, controlled exposure studies can assess the direct causal effect of PM_2.5_ exposure by comparing an individual’s response following clean air and PM_2.5_ exposures. PM_2.5_ related effects observed in these exposure studies do not need to be disassociated from the impacts of other pollutants and confounders, providing more direct evidence of the potential of these particles to exert pulmonary and extra-pulmonary effects [13–16]. However, nearly all these studies have been conducted at realistic but high levels of PM_2.5_ (typically above 100 μg/m^3^). This has led to concern that the biological pathways changed at these elevated concentrations may not be relevant at lower concentrations.

In this study, we measured the cardiopulmonary changes in healthy young volunteers exposed acutely to levels of PM_2.5_ near the 24-h US National Ambient Air Quality Standard (NAAQS) for PM_2.5_ of 35 μg/m^3^. Subjects underwent intermittent exercise to regulate exposure, which may simulate light intensity exercise outside. In panel studies involving physical activity, air pollution was associated with reductions in cardiorespiratory biomarkers [12, 17, 18]. We examined lung function, systemic changes in the blood, and the electrophysiology following 4-h PM_2.5_ exposures. We hypothesized that the same pathways activated by elevated levels of PM_2.5_ would also be activated at ambient levels found in many urban areas.

## Methods

### Study population

Healthy adult subjects, aged 18–35 years, were recruited from the central North Carolina area between February and October 2017. Inclusion criteria included the ability to complete the exercise regimen without reaching 80% of the predicted maximal heart rate [19] while maintaining an oxygen saturation of ≥92%, a normal baseline electrocardiography (ECG), and normal lung function (FVC and FEV_1_ ≥ 80% of that predicted for sex, ethnicity, age, and height) [20]. Subjects were excluded if they were smokers (frequent smokers and those with a history of > 5 pack years) or had acute or chronic illnesses such as asthma, diabetes, cancer, or any autoimmune abnormalities. Prior to enrollment, subjects were informed of the study procedures and risks and informed consent was obtained. The study protocol and consent forms were approved by the University of North Carolina at Chapel Hill Institutional Review Board and the US Environmental Protection Agency Human Subjects Research Review Office. The study has been registered as a clinical trial (ClinicalTrials.gov # NCT03232086). The study enrolled 21 subjects; however, the data for 20 subjects are presented as one subject did not complete both exposures.

### Study design and exposure to PM_2.5_

This was a randomized, double-blind, crossover study. Subjects received two 4-h exposures, one to clean air (control exposure) and one to concentrated PM_2.5_, with intermittent exercise, on two separate occasions separated on average by 38 days, to limit cross exposure effects, at the EPA Human Studies Facility. The airshed around the facility is urban and primarily driven by mobile sources (e.g. traffic). The exposure order was randomized by subject. Subjects were asked to refrain from consuming alcohol and caffeine 24 h prior to all exposures. The 4-h exposure was administered as a 2-h exposure, followed by a 30-min period to record blood pressure, perform a symptom questionnaire, and have a restroom break, then a second 2-h exposure. During exposures subjects alternated between cycles consisting of 15 min of exercise on a stationary recumbent bicycle and 15 min of rest. Tension on the exercise bike was adjusted for subjects to achieve the target ventilation rate of 20 L/min/m^2^ body surface area, which would enable subjects to receive approximately the same dose of PM_2.5_ [21]. Spirometry, venipuncture, and Holter monitor measures of heart rate variability and repolarization were performed immediately prior to exposure (pre-exposure), 1 h post exposure (post-exposure), and 20 h post exposure (follow-up exposure) as previously described [22–24].

Exposure to filtered air and concentrated PM_2.5_ particles was conducted as described previously [22] by concentrating ambient air from around the Human Studies Facility with a 2-stage aerosol Harvard concentrator. The target exposure was 35 μg/m^3^, which corresponds to the 24 h NAAQS. Due to variation of ambient air quality over the course of ambient air quality over the 4 h and day to day, the concentrator was able to achieve an average concentration of 37.8 μg/m^3^ (SD 6.5). Table S1 shows the actual exposure concentrations for each subject. All exposures began within 30 min of 0930 h to control for diurnal variations in physiologic response.

### Exposure methodology regarding concentrated PM_2.5_ exposure

Concentrated particles were generated by drawing ambient air from above the roof of the US Human Studies Facility (Chapel Hill, NC) and passing the air through a two-stage aerosol concentrator which produces up to a 30-fold increase in particle number and mass. Particles larger than about 2.5 μm were excluded by a size-selective inlet from entering the concentrator at the rooftop intake. During the particle concentrating process, ambient air pollution gases were diluted. Air temperature and humidity were monitored and maintained to ensure proper operation of the concentrator. The chamber exposure atmosphere averaged 40% ± 10% relative humidity and approximately 22 ± 2 °C. The concentration of particles delivered to the chamber varies depending on the levels of naturally occurring particles in the Chapel Hill, NC air as well as meteorological and other factors. Therefore, particle concentrations in the chamber were monitored continually and the particle concentration diluted with clean air as needed to achieve the target concentration range of 35–50 μg/m3. Exposures were monitored in real time through instrumentation and the actual concentration of concentrated particles delivered was determined retrospectively by filter gravimetry.

### End point measurements

#### Spirometry

Pulmonary function was measured with a SensorMedics Vmax system (VIASYS, Conshohocken, PA). Forced vital capacity (FVC), FEV_1_, mid-expiratory flow rate (FEF 25–75%), and peak expiratory flow (PEF) were measured pre-exposure, post-exposure, and during follow-up as described earlier [21, 25].

#### Cellular and soluble blood components

Venous blood was collected pre-exposure, post-exposure, and during follow-up for each exposure. Basic blood chemistry and complete blood count with differential counts by cell type were measured by LabCorp (Burlington, NC) on the day the blood was drawn. Plasma and serum were also frozen at − 80 °C for later analysis. Basic blood chemistry endpoints measured included cholesterol (mg/dL), triglycerides (mg/dL), low-density lipoproteins (LDL, mg/dL), very low-density lipoproteins (VLDL, mg/dL), and high-density lipoproteins (HDL mg/dL). Complete blood count included white blood cells (WBC, 10^3^/μL), red blood cells (RBC, 10^3^/μL), platelets (10^3^/μL), hemoglobin (g/dL), neutrophils (%), lymphocytes (%), and hematocrit (%). Analyses were performed on the Meso Scale Discovery multiplex assay system (Meso Scale Diagnostics, LLC, Rockville, MD) in two separate kits: one for pro-inflammatory mediators: interleukin 1-beta (IL-1β), IL-6, IL-8, and tumor necrosis factor-alpha (TNF-α) and another for vascular injury: CRP, serum amyloid A (SAA), soluble intercellular adhesion molecule-1 (sICAM-1), soluble vascular cell adhesion molecule-1 (sVCAM-1). All other assays (d-dimer, tissue plasminogen activator (tPA), von Willebrand Factor (vWF) and plasminogen activator inhibitor 1 (PAI-1)) were established using the Multiarray plates as per manufacturer’s instructions.

#### Ambulatory electrocardiography (ECG) measurements

Heart rate variability (HRV) and repolarization endpoints were measured using an ambulatory electrocardiographic monitor as described previously [22, 26]. Briefly, continuous ambulatory electrocardiograms (ECGs) were collected using a Mortara H12+ 12-Lead ECG Recorder (Mortara Instrument Co., Milwaukee, WI). Subjects rested in a quiet dark room for 20 min and Holter parameters were measured from the next 5 min. We calculated high frequency (HF, 0.15–0.4 Hz), low frequency (LF, 0.04–0.15 Hz) and total power (0–0.4 Hz) in absolute units (LF and HF components were normalized to account for changes in heart rate). Time-domain parameters measured included heart rate (min, max, mean), standard deviation of NN intervals (SDNN), and percentage of successive RR intervals that differ by more than 50 ms (pNN50). Cardiac repolarization endpoints measured included the duration of the QT interval, heart rate corrected QT (QTc), P-wave, QRS complex, and T-wave. P-wave duration is a measure of atrial depolarization and contraction. QRS complex is a measure associated with ventricular depolarization. QTc is the interval where ventricles depolarize and repolarize. T-wave is a measure of ventricular repolarization.

### Statistical analysis

We assessed lung function, blood markers, and HRV measurements immediately before, 1 h after, and 20 h after each exposure. For the analysis, we normalized post-exposure and follow-up exposure values by the corresponding pre-exposure value and compared the percent point difference between filtered air exposure and PM_2.5_ exposure. Linear mixed effects models with random intercept for subject were used to measure differences in response between the filtered air and PM_2.5_ exposures (R statistical software, version 3.6.2, package *lme4*) [27]. We used a subject level random intercept to account for the subject-level variability and estimated slope parameters to characterize the expected change in response as a function of PM_2.5_ concentration. Estimated slopes are presented as the percent point differences in normalized values (95%CI) per 37.8 μg/m^3^ increase in PM_2.5_ exposure (37.8 μg/m^3^ corresponds to the mean concentration during PM_2.5_ exposures). Sex specific changes were not primary effects of interest and are reported here when significant. The study was powered based on primary endpoints that included forced expiratory volume in 1 s (FEV_1_), blood neutrophils, duration of the QT interval, and C-reactive protein (CRP). The power calculation based on the primary endpoints determined that 20 subjects was needed for adequate power (80%). Statistical significance was determined at *p* < 0.05.

## Results

### Study population and PM_2.5_ exposure

A total of 21 healthy subjects were recruited, and 20 (7 females and 13 males) completed the study. Most self-classified as white (*n* = 14) and had a mean age of 25.3 years (Table 1, Table S1).

**Table 1:** Descriptive statistics of the study population (*n* = 20)

Demographic variable Mean (+/- SD)	Mean (+/- SD)
Sex (%)	
Male	65%
Female	35%
Race (%)	
White	70%
Black	20%
Mixed (W/B)	10%
Age (years)	25.3 ± 4.0
BMI (kg/m^2^)	25.5 ± 3.6

PM_2.5_ concentration in the chamber is measured on a continuous scale and varies from subject to subject depending on the outdoor PM concentration that day. Table 1 shows the average chamber concentrations during the filtered air and PM_2.5_ exposures. PM_2.5_ concentrations for filtered air and PM_2.5_ exposures averaged 2.2 μg/m^3^ (range: − 0.80 - 10.45 μg/m^3^) and 37.8 μg/m^3^ (range: 25.8–52.9 μg/m^3^), respectively (Table 2).

**Table 2:** Average PM_2.5_ concentration (± standard deviation) for clean air and concentrated PM_2.5_ exposures (μg/m^3^)

Exposure Treatment	Mean (± SD)
Clean air	2.1 ± 2.6
Concentrated PM_2.5_	37.8 ± 6.5

### Near ambient levels of PM_2.5_ decreased lung function

**Fig. 1:**
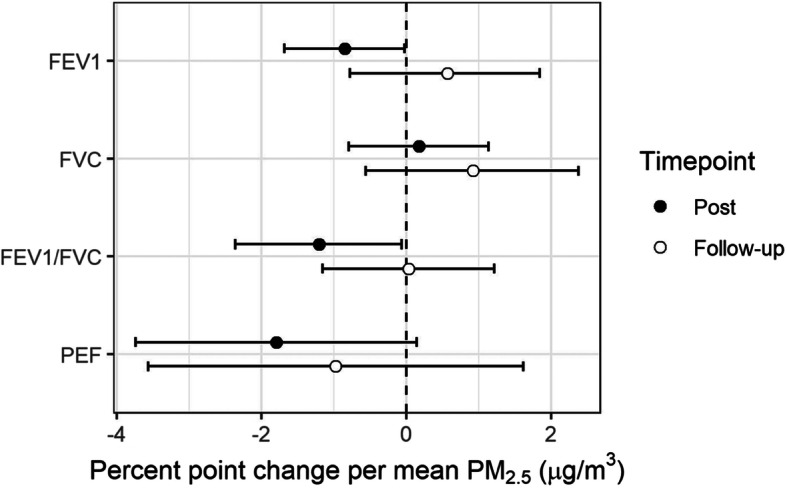
Mean percent point change in lung function (95%CI): 1 h after exposure (Post) and approximately 20 h after exposure (Follow-up). Mean change is expressed per 37.8 μg/m^3^ of PM_2.5_, which corresponds to average concentrated PM_2.5_ exposure across subjects

Elevated PM_2.5_ was associated with decreased lung capacity and reduced pulmonary function. One hour after PM_2.5_ exposure, FEV_1_ and PEF were marginally decreased by 0.8% (95%CI: 0.03–1.7%, *p* = 0.054) and 1.8% (95%CI: − 0.1–3.7%, *p* = 0.077), respectively. FEV_1_/FVC was significantly decreased by 1.2% (95%CI: 0.1–2.4%, *p* = 0.048) 1 h post-exposure (Fig. 1, Table 3, Table S2). Sex specific changes were observed with the decrease in FEV_1_ 1 h post-exposure, where in males the FEV_1_ significantly decreased by 1.4% (95%CI: 0.4–2.5%, *p* = 0.02) (Fig. 1, Table 3, Table S2). As expected, all lung function measures had returned to baseline by 20 h post-exposure.

**Table 3:** Changes in blood chemistry, cellular, HRV, inflammatory, lung function, and repolarization measures (95%CI): 1 h after exposure (Post) and approximately 20 h after exposure (Follow-up). Mean change is expressed per 37.8 μg/m^3^ of PM_2.5_, which corresponds to average concentrated PM_2.5_ exposure across subjects

Group	Endpoint	Post	Follow-up
Blood Chemistry	Cholesterol (mg/dL)	1.4 (− 3.0, 5.9)	0.6 (− 3.1, 4.4)
	HDL (mg/dL)	− 0.4 (− 5.3, 4.6)	− 1.4 (− 6.3, 3.6)
	LDL (mg/dL)	5.5 (− 3.7, 14.6)	4.3 (− 1.9, 10.8)
	Triglycerides (mg/dL)	− 14.3 (− 40.5, 13.3)	− 17.3 (− 41.2, 6.6)
	VLDL (mg/dL)	− 12.9 (− 38.4, 13.9)	−16.8 (− 41.2, 7.6)
	D-Dimer (ng/mL)	10.7 (− 12.1, 33)	− 4.4 (− 21.5, 12.6)
	LDH (IU/L)	− 6.9 (− 13.3, − 0.5)	− 11.2 (− 17.4, − 5.0)
	PAI-1 (ng/mL)	−1.2 (− 16.2, 13.6)	7.0 (− 15.9, 29.3)
	Plasminogen (%)	2.2 (− 5.1, 9.6)	1.7 (− 6.9, 10.4)
	Protein (g/dL)	− 0.2 (− 3.2, 2.8)	0.1 (− 1.9, 2.2)
	tPA (ng/mL)	3.9 (− 3.8, 11.4)	− 3.5 (− 13.1, 5.8)
	vWF (%)	− 6.9 (− 23.0, 9.2)	6.0 (− 7.5, 19.5)
Cellular	Basophils (%)	− 3.6 (− 51.2, 44.0)	− 0.4 (− 38.1, 40.4)
	Eosinophils (10^3^ cells/uL)	− 38.4 (− 146.2, 70.8)	− 4.5 (− 19.6, 11.5)
	Eosinophils (%)	1.2 (− 15.8, 18.3)	2.1 (− 16.9, 21.7)
	Lymphocytes (%)	2.1 (− 3.5, 7.7)	6.3 (− 0.9, 14.2)
	Monocytes (10^3^ cells/uL)	− 2.2 (− 13.8, 9.1)	− 2.6 (− 14.2, 8.3)
	Monocytes (%)	0.8 (− 11.1, 12.3)	2.9 (− 8.1, 13.6)
	Neutrophils (%)	−6.5 (− 13.9, 0.8)	−5.7 (− 11.1, − 0.6)
	Platelets (10^3^ cells/uL)	− 0.8 (− 6.3, 4.8)	− 0.5 (− 4.7, 3.8)
	RBC (10^3^ cells/uL)	− 0.5 (− 2.5, 1.4)	− 1.6 (− 3.2, 0.0)
	WBC (10^3^ cells/uL)	3.3 (− 11.8, 18.5)	−4.8 (− 14.1, 4.5)
HRV	HF/LF	12.7 (− 17.4, 42.7)	17.0 (− 31.3, 65.2)
	HFn	8.6 (− 6.4, 23.7)	13.0 (− 12.8, 38.7)
	LFn	−0.7 (− 19.1, 16.6)	− 13.3 (− 31.3, 4.8)
	pNN50 (# of NN intervals)	2.0 (− 33.8, 38.0)	154.5 (− 178.2, 487.2)
	SDNN (ms)	−6.6 (− 25.2, 12.0)	10.6 (− 21.8, 43.0)
Inflammatory	CRP (ng/mL)	9.1 (1.0, 17.2)	22.8 (−2.9, 48.7)
	IL-1b (pg/mL)	−3.0 (− 14.7, 8.7)	1.9 (−8.7, 13.1)
	IL-6 (pg/mL)	12.7 (−9, 34.4)	−3.0 (− 17.3, 11.5)
	IL-8 (pg/mL)	−1.5 (−9.9, 7.0)	0.8 (− 13.2, 14.8)
	sICAM (ng/mL)	10.7 (4., 17.5)	6.5 (−1.7, 14.7)
	SAA (ng/mL)	8.7 (1.3, 16.1)	34.6 (13.1, 56.1)
	sVCAM (ng/mL)	6.6 (1.3, 12.0)	2.5 (−3.9, 8.9)
	TNF-a (pg/mL)	−1.4 (−6.1, 3.3)	0.2 (−7.1, 7.5)
Lung function	FEF 25–75%	−1.6 (−4.3, 1.1)	0.6 (−2.4, 3.5)
	FEV_1_	−0.8 (−1.7, 0.0)	0.6 (− 0.8, 1.8)
	FEV_1_/FVC	−1.2 (− 2.4, − 0.1)	0.0 (− 1.2, 1.2)
	FVC	0.2 (− 0.8, 1.1)	0.9 (− 0.6, 2.4)
	PEF	− 1.8 (−3.7, 0.1)	− 1.0 (− 3.6, 1.6)
Repolarization	Max HR (beats per min)	−3.3 (−9.3, 2.6)	1.9 (−6.5, 10.2)
	Mean HR (beats per min)	− 1.9 (−8.6, 4.5)	−0.4 (−6.2, 5.5)
	Min HR (beats per min)	−0.9 (−9.3, 7.6)	−1.7 (− 9.7, 6.2)
	P-wave (ms)	10.5 (4.0, 17.1)	−5.7 (− 20, 8.7)
	QRS complex (ms)	2.2 (−1.6, 6.0)	−4.6 (−9.9, 0.7)
	QT interval (ms)	−0.1 (−1.7, 1.5)	0.4 (−2.2, 3.1)
	QTc interval (ms)	−0.3 (−1.1, 0.6)	0.1 (−1.6, 1.7)
	T-wave (ms)	−0.9 (−9.7, 7.9)	−6.1 (−13.2, 1.2)

### Near ambient levels of PM_2.5_ resulted in vascular inflammation and acute phase injury

**Fig. 2:**
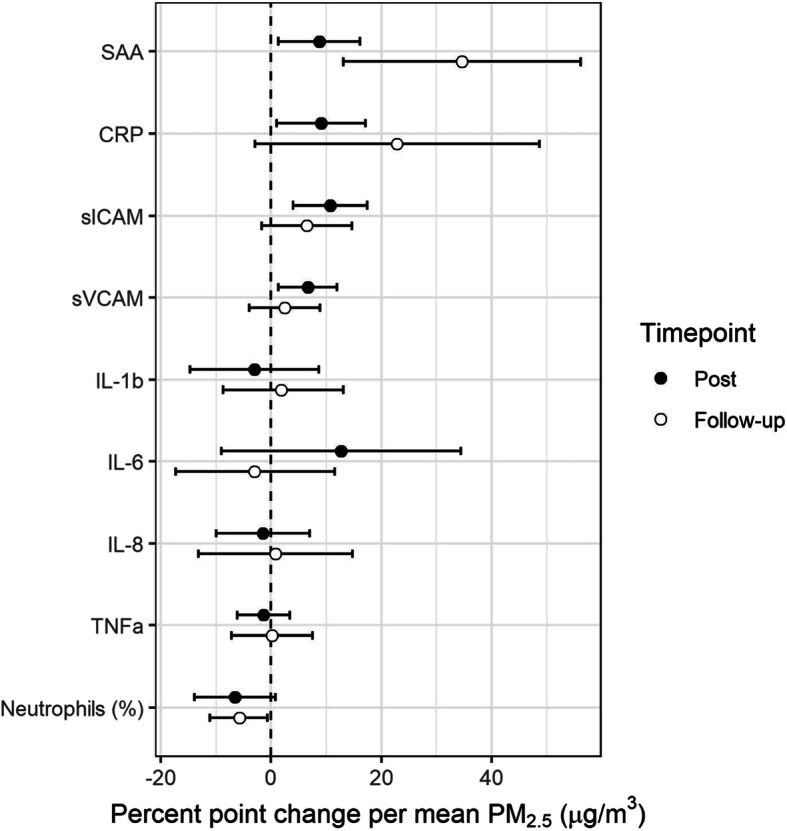
Mean percent point change in systemic inflammation markers (95%CI): 1 h after exposure (Post) and approximately 20 h after exposure (Follow-up). Mean change is expressed per 37.8 μg/m^3^ of PM_2.5_, which corresponds to average concentrated PM_2.5_ exposure across subjects

PM_2.5_ exposure was associated with increased serum levels of several inflammatory markers. Relative to filtered air, acute phase inflammation markers SAA and CRP were significantly increased 1 h after the average PM_2.5_ exposure by 8.7% (95%CI: 1.3–16.1%, *p* = 0.027), 9.1% (95%CI: 1.0–17.2%, *p* = 0.034), respectively. SAA and CRP were further elevated 20 h after exposure where SAA was 34.6% (95%CI: 13.1–56.1%, *p* = 0.004) higher and CRP was 22.8% (95%CI: − 2.9-48.7%, *p* = 0.092) higher (marginal) than pre-exposure measurements. Additionally, relative to measures taken before exposure, percent neutrophils were decreased by 6.5% (95%CI: − 0.8-13.9%, *p* = 0.091), although not significant, and significantly decreased by 5.7% (95%CI: 0.6–11.1%, *p* = 0.040) 1 h and 20 h after PM_2.5_ exposure, respectively (Fig. 2, Table 3, Table S2).

An inflammatory response was also observed through increases in endpoints typically stimulated by inflammatory cytokines. Compared to filtered air, sICAM and sVCAM were significantly increased 1 h after the average PM_2.5_ exposure by 10.7% (95%CI: 4.0–17.5%, *p* = 0.003) and 6.6% (95%CI: 1.3–12.0%, *p* = 0.022), respectively (Fig. 2, Table 3, Table S2). Among the inflammatory cytokines which are upstream of sICAM and sVCAM, we observed a 12.7% increase (95%CI: − 9.0-34.4%, *p* = 0.26) in IL-6 1 h after exposure. However, that change was not statistically significant. There were also no observed statistically significant changes in other inflammatory cytokines including IL-1b, IL-8, and TNF-α (Fig. 2, Table 3, Table S2).

### Near ambient levels of PM_2.5_ resulted in changes in HRV and cardiac repolarization

**Fig. 3:**
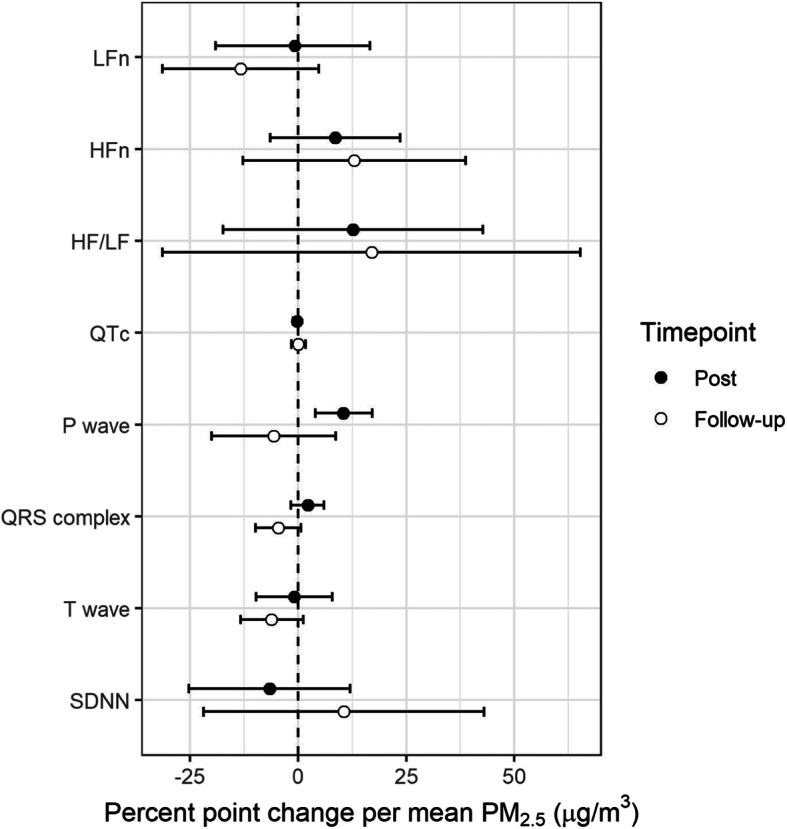
Mean percent point change in HRV and cardiac repolarization measures (95%CI): 1 h after exposure (Post) and approximately 20 h after exposure (Follow-up). Mean change is expressed per 37.8 μg/m^3^ of PM_2.5_, which corresponds to average concentrated PM_2.5_ exposure across subjects

No significant associations were observed between PM_2.5_ and the HRV time domain measurements SDNN and pNN50. When considering effects within strata by sex, SDNN increased by 23.9% (95%CI: 8.6–41.1%, *p* = 0.02) in females and decreased by 26.2% (95%CI: 1.9–50.6%, *p* = 0.046) in males 1 h after PM_2.5_ exposure. HRV frequency domain parameters (nHF, nLF, HF/LF) measured 1 h or 20 h post exposure did not have significant associations with PM_2.5_ (Fig. 3, Table 3, Table S2).

Cardiac repolarization measures including the duration of the P-wave, QRS complex, QT interval (QTc), and T-wave were altered by PM_2.5_ exposure. P-wave duration increased by 10.5% (95%CI: 4.0–17.1%, *p* = 0.005) 1 h post exposure and the QRS complex decreased marginally by 4.6% (95%CI: − 0.7-9.9%, *p* = 0.095) 20 h post exposure to PM_2.5_. Changes in repolarization measures were more pronounced in men where 1 h after PM_2.5_ exposure the P-wave and QRS complex, increased by 15.4% (95%CI: 8.7–22.3%, *p* = 0.001) and 5.4% (95%CI: 1.0–9.5%, p = 0.02), respectively. QTc and T-wave measures did not change for the whole group, but in females QTc was decreased by 1.6% (95%CI: 0.5–2.9%, *p* = 0.03) 1 h after exposure and T-wave was decreased by 17.4% (95%CI: 6.9–27.6%, *p* = 0.01) 20 h after exposure (Fig. 3, Table 3, Table S2).

### Near ambient levels of PM_2.5_ resulted in changes in markers of cell injury and red blood cell function

**Fig. 4:**
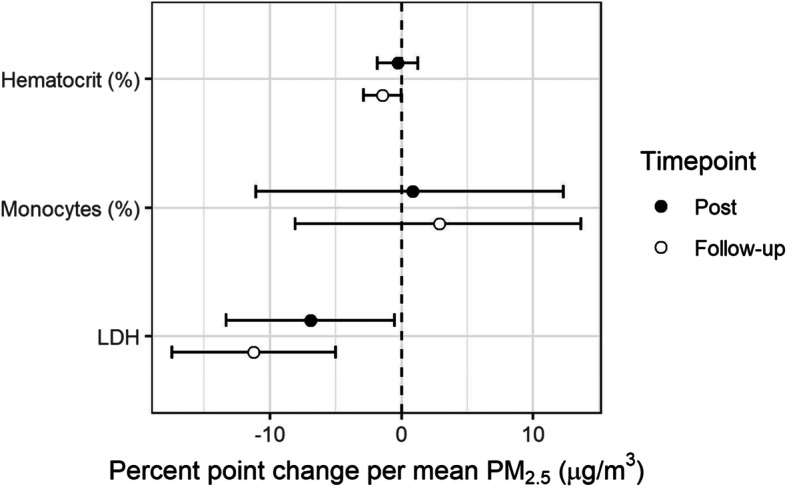
Mean percent point change in blood chemistry and lipids measures (95%CI): 1 h after exposure (Post) and approximately 20 h after exposure (Follow-up). Mean change is expressed per 37.8 μg/m^3^ of PM_2.5_, which corresponds to average concentrated PM_2.5_ exposure across subjects

Elevated PM_2.5_ was associated with decreases in multiple cellular measures. LDH, a marker of cell injury was decreased by 6.9% (95%CI: 0.5–13.3%, *p* = 0.04) 1 h post and by 11.2% (95%CI: 5.0–17.4%, p = 0.001) 20 h post. Percent hematocrit was 1.4% (95%CI: 0.01–2.9%, *p* = 0.058) lower (marginally) than pre-exposure measurements 20 h after PM_2.5_ exposure (Fig. 4, Table 3, Table S2). We did not observe significant changes in the other systemic blood or lipid markers measured (Table 3).

## Discussion

This study is the first to provide direct evidence of detrimental health impacts related to short-term low-concentration fine particulate matter exposure in healthy young volunteers. PM_2.5_ concentrations in this study were close to the daily US NAAQS standard and are applicable to a large proportion of the general US population. Over 75 million individuals reside in a county that exceeds the average PM_2.5_ concentration in this study for 1 day annually, and over 54 million reside in a county that exceeds this average for 7 or more days [28]. At PM_2.5_ concentrations near the daily US NAAQS, we found alterations in biomarkers of vascular injury, cardiac electrophysiology, and lung function, indicating that similar pathways appear to be altered at both high and low PM_2.5_ concentrations.

In healthy young adults exposed to low PM_2.5_ concentrations for 4 h, we observed 7 to 11% increases in vascular inflammation markers, SAA, CRP, sICAM, and sVCAM, 1 h after exposure. One hour after exposure, we also observed 1 to 2% decreases in lung capacity (FEV_1_ and PEF) and lung function (FEV_1_/FVC). Additionally, neutrophils and lactase dehydrogenase were decreased 6–11% both 1 h and 20 h post exposure. To our knowledge, this is the first clinical study to examine repolarization measures at levels of PM_2.5_ near the current daily PM_2.5_ US NAAQS. Previous short-term controlled exposures to concentrated air particles were done at average concentrations ranging from 89 to 278 μg/m^3^. In models with all individuals, we observed changes in cardiac repolarization including a 10.5% increase in P-wave 1 h after exposure, and a 4.6% decrease in QRS-wave 20 h after exposure. These changes were pronounced in models containing only males. Differences by sex were also observed with other cardiac repolarization and time domain measurements. In the female strata, QTc decreased by 1.6% 1 h after exposure and T-wave decreased by 17.4% 20 h after exposure. With the time domain measurement, SDNN increased 23.9% in females and decreased 2.2% in male strata 1 h post. The reported results suggest that there could be impacts to depolarization and repolarization in some populations that could lead to arrhythmia. However, we cannot make strong conclusions regarding differences by sex due to the small sample size. Further research is necessary to examine potential sex related differences. Importantly, the cardiopulmonary effects observed here are similar to studies with higher PM_2.5_ exposures, providing evidence that the pathways altered at high-dose exposures are also impacted at low-dose exposures.

Associations with a pro-inflammatory response have been observed previously in other clinical studies [13, 24, 25, 29–33]. In studies with a similar exposure design, this response has been observed through PM related increases in TNF-α [13], inflammatory proteins (CRP and SAA) [29], fibrinogen [25, 32], and plasminogen activator and endothelin-1 levels [33]. Evidence of systemic and localized inflammation has also been observed in laboratory studies through increases in cellular adhesion molecules in an in vitro study [34] and elevated TNF-α and IL-6 levels in lung tissue [35–38]. In this study, we evaluated effects of systematic inflammation from short term exposure, but did not observe significant associations with IL-6, IL-8, IL-1b, and TNF-α; however, we did observe a trend with IL-6 being elevated post exposure (13%). Previously, IL-6 has been observed to be elevated with long-term PM_2.5_ exposures [39]. We also observed decreases in white blood cells following acute PM_2.5_ exposure, similar to another controlled human exposure study with healthy volunteers [40]. The observed decrease in neutrophils in these studies may reflect a movement of white blood cells from large vessels to smaller arteries and capillaries. Indeed, prior work has observed significant increases in neutrophils in lavage samples, suggesting localized inflammation in lung tissue [25, 31, 32]. A reduction in lactate dehydrogenase was also observed in this study and while we are not sure on the mechanism, a similar decrease was observed in prior work [40].

Evidence of lung function impairments in association with long-term PM_2.5_ levels has been observed in environmental studies, but not with acute exposures in clinical studies. Lung function changes were not observed in our previous study that utilized a 2 h exposure and we hypothesize that the differences in observations in the current study are due to the longer exposure time. To our knowledge, this is the first study to observe potential obstructive impacts, via a significant decrease in the FEV_1_/FVC ratio, following a short-term exposure to moderate levels of PM_2.5_. Decreases in lung capacity have also been observed previously in ecological studies of children [41, 42], asthmatic children [43], and adults [44–47] through reductions in forced expiratory volume in 1 s (FEV_1_), forced vital capacity (FVC), and peak expiratory flow (PEF). Our results were comparable to a previous study conducted in healthy adults that had higher ambient PM_2.5_ concentrations and considered interactions with temperature. At temperatures above 20 °C, elevated PM_2.5_ was associated with a 1.5–1.9% decrease in FEV_1_ [46]. The percent change in FEV_1_ observed in this study was lower than the 5–8% decrease in FEV_1_ observed in an ecological study in children with asthma [43]. Acute decreases in lung capacity may result from airway inflammation related to reactive oxygen species activation of MAPK and NF-κB signaling pathways or from an exacerbated Th immune response [48, 49].

In addition to respiratory impacts, cardiovascular effects have been observed in prior research through changes in heart rate variability and cardiac repolarization measures [13, 22, 24, 31]. PM associated changes to the frequency domain and repolarization measures have not been observed consistently in prior clinical studies. Studies reporting HRV differences have observed increased high and low frequency measures [24], a reduction in the high-frequency/low-frequency ratio [22], and a decreased SDNN [31]. In this study, HRV measures (HF, LF, HF/LF ratio, pNN50, SDNN) were not observed to be impacted. Additionally, no changes in ventricular depolarization (QRS complex) were observed in this study, similar to a previous study [29]. However, cardiac repolarization changes including ventricular repolarization have been observed in studies with controlled PM exposures through prolongation of the QT interval [22, 24, 29]. Exposures in clinical studies may be too brief to observe differences in HRV endpoints, as significant changes have been observed in environmental studies assessing associations with long-term exposures [50–52].

Physical activity has a well-established benefit to general and cardiovascular health [53, 54], but it can also increase personal exposure through elevated ventilation rates thus presenting a more complex relationship [55]. Although there is not a consensus on the extent that physical activity may moderate air pollution related health outcomes [56], previous studies have reported changes in lung function, inflammation, and HRV in association with the short-term effects of air pollution exposure in individuals undergoing physical activity [18, 55, 57, 58]. In panel and cross-over studies, short-term exposure to traffic related air pollution, diesel, and ultrafine particles was associated with reduced lung function, observed through decreases in FEV1, FVC, and PEF and increased fraction of exhaled nitric oxide [12, 17, 55, 57, 58] and altered HRV, observed through decreases in HF and QT interval [17, 18, 55, 59, 60]. There is also evidence for systemic and localized lung inflammation [58]. In current study, physical activity was used to standardize dose between subjects and was not evaluated as an effect modifying factor. In that context, our results suggest that low concentrations of PM_2.5_ may acutely increase inflammation and reduce lung function in healthy young adults undergoing moderate physical activity.

There are a number of reasons why we hypothesize effects were observed in this study. First, raised ventilation rate related to the moderate physical activity would deliver a greater dose to subjects. The target ventilation rate in this study was greater than prior studies where subjects were exposed at rest [23, 32]. Secondly, the exposure time employed was twice that of many prior studies using concentrated air particles [15, 22, 23, 32, 40]. The combination of a greater ventilation rate and longer exposure time would lend to a comparable 2 h exposure of approximately 2–4 times (75–150 μg/m^3^), depending on study design. In this exposure range, inflammation, cardiac repolarization, and decreased pulmonary function have been observed in other chamber studies involving concentrated air particles [15, 22, 23, 32, 40]. Cardiopulmonary changes have also been observed in studies with similar exposure methods and ultrafine particles [31, 55, 61, 62]. Important to note that direct comparison with other types of air pollution like wood smoke and diesel are a challenge as these types of air pollution are chemically different.

This study also had some limitations. Outside of lung function and HRV, our observations for blood endpoints including inflammation and cellular changes were limited to systemic impacts and do not provide information on localized effects in certain tissues or organs. Additionally, exposure to air pollutants or other toxicants in the day(s) preceding the subject’s visit to the facility could have impacted their response and were not specifically controlled for in this study. As this study was conducted with young, healthy adults it may not be generalizable to other populations. We recognize that the sample size combined with the number of secondary endpoints present a limitation and could lead to false positive observations by chance. Using the Bonferroni correction would lead to no significant findings. However, there were also strengths. The randomized crossover study design reduced the influence of confounding because subjects serve as their own control. Another strength was that by controlling a subject’s ventilation rate, based on body surface area, subjects received approximately the same internal dose of PM_2.5_. Lastly, gaseous pollutants such as ozone and nitrogen dioxide or vapors from air toxics were removed during the concentration process unlike in observational studies where exposures are experienced simultaneously. As such, the observed effects could be attributed directly to PM_2.5_.

## Conclusion

This study builds on previous clinical exposure research by assessing PM-related effects on cardiopulmonary endpoints in healthy young adults at low PM_2.5_ concentrations, near the current US NAAQS standard, whereas previous studies used concentrations in the range of 89 to 278 μg/m^3^. This is a major strength as observations would be directly attributed to PM exposures that are relevant to low-dose exposure, which occurs in many regions in the US. We observed changes in lung function, markers of systemic inflammation, and vascular injury. Data from this study suggests that low-level PM_2.5_ concentrations near the US NAAQS daily standard induce similar alterations in biomarkers of vascular injury and lung function in young healthy adults.

## Supplementary Information

**Additional file 1:** **Table S1.** Subject level demographic and PM_2.5_ concentration data.

**Additional file 2:** **Table S2.** Mean percent point change and differences in inflammatory, HRV and cardiac repolarization, lung function, blood chemistry, and lipids measures (95%CI): 1 h after exposure (Post) and approximately 20 h after exposure (Follow-up). Mean change is expressed per 37.8 μg/m^3^ of PM_2.5_, which corresponds to average concentrated PM_2.5_ exposure across subjects. **Table S3.** Mean values for inflammatory, HRV and cardiac repolarization, lung function, blood chemistry, and lipids measures for air and PM_2.5_ exposures: Pre-exposure, 1 h after exposure (Post) and approximately 20 h after exposure (Follow-up).

## Data Availability

The datasets used and/or analyzed during the current study are available from the corresponding author on reasonable request.
