# Supplementary material for: Low levels of fine particulate matter increase vascular damage and reduce pulmonary function in young healthy adults
Source: Part Fibre Toxicol. 2020 Nov 16;17:58. doi: 10.1186/s12989-020-00389-5 (PMC7670817; doi:10.1186/s12989-020-00389-5)
Supplement: Supplementary file 1 — Additional file 1: Table S1. Subject level demographic and PM2.5 concentration data. [file 12989_2020_389_MOESM1_ESM.docx]

Supplementary Materials

Table S1. Subject level demographic and PM_2.5_ concentration data.

| Subject | Sex | Age (years) | BMI | Race | PM concentration (μg/m^3^) | |
| --- | --- | --- | --- | --- | --- | --- |
|  |  |  |  |  | Air | Concentrated |
| 1 | Male | 22 | 24.6 | W | 5.3 | 28.9 |
| 2 | Female | 35 | 21.9 | W | 4.0 | 39.0 |
| 3 | Female | 27 | 29.0 | Mixed AA/W | 1.8 | 39.9 |
| 4 | Male | 22 | 21.6 | W | 2.9 | 40.6 |
| 5 | Male | 28 | 23.8 | W | 2.8 | 41.5 |
| 6 | Male | 31 | 29.1 | B | 4.3 | 36.1 |
| 7 | Female | 25 | 25.4 | W | 0.3 | 44.5 |
| 8 | Male | 24 | 32.3 | B | 1.9 | 34.4 |
| 9 | Female | 20 | 23.5 | W | 0.3 | 46.4 |
| 10 | Female | 24 | 23.0 | Mixed AA/W | 0.0 | 31.7 |
| 11 | Male | 27 | 23.6 | W | 1.7 | 27.3 |
| 12 | Male | 26 | 25.3 | W | 10.4 | 52.9 |
| 13 | Male | 33 | 22.9 | W | 1.7 | 34.6 |
| 14 | Female | 25 | 33.6 | B | 3.8 | 34.0 |
| 15 | Male | 22 | 28.1 | W | 0.0 | 25.8 |
| 16 | Male | 23 | 23.3 | W | 1.1 | 40.3 |
| 17 | Female | 25 | 29.7 | B | 0.3 | 38.6 |
| 18 | Male | 21 | 20.1 | W | 0.0 | 40.3 |
| 19 | Male | 24 | 23.8 | W | 1.0 | 39.0 |
| 20 | Male | 22 | 26.0 | W | 0.4 | 40.4 |
| Mean ± SD | | 25.3 ± 4.0 | 25.5 ± 3.6 |  | 2.2 ± 2.6 | 37.8 ± 6.5 |

Table S2. Mean percent point change and differences in inflammatory, HRV and cardiac repolarization, lung function, blood chemistry, and lipids measures (95%CI): 1 hr after exposure (Post) and approximately 20 hr after exposure (Follow-up). Mean change is expressed per 37.8 μg/m^3^ of PM_2.5_, which corresponds to average concentrated PM_2.5_ exposure across subjects.

(separate file because of length)

Table S3. Mean values for inflammatory markers, HRV and cardiac repolarization, lung function, blood chemistry, and lipids measures for air and PM_2.5_ exposures: Pre-exposure, 1 hr after exposure (Post) and approximately 20 hr after exposure (Follow-up).

(separate file because of length)
